# Germination of *Pisum sativum* L. Seeds Is Associated with the Alternative Respiratory Pathway

**DOI:** 10.3390/biology12101318

**Published:** 2023-10-09

**Authors:** Lénia Rodrigues, Amaia Nogales, João Nunes, Leonardo Rodrigues, Lee D. Hansen, Hélia Cardoso

**Affiliations:** 1 MED—Mediterranean Institute for Agriculture, Environment and Development & CHANGE—Global Change and Sustainability Institute, Institute for Advanced Studies and Research, University of Évora, Pólo da Mitra, Ap. 94, 7006-554 Évora, Portugal; liar@uevora.pt; 2 IRTA Institute of Agrifood Research and Technology, Sustainable Plant Protection Programme, Centre Cabrils, Ctra. Cabrils Km 2, 08348 Cabrils, Spain; amaia.nogales@irta.cat; 3 School of Sciences and Technology, University of Évora, Pólo da Mitra, Ap. 94, 7006-554 Évora, Portugal; joao.m.nunes15@gmail.com (J.N.); leoabraaorodrigues@gmail.com (L.R.); 4 Department of Chemistry and Biochemistry, Brigham Young University, Provo, UT 84602, USA; ldhansen@chem.byu.edu; 5 MED—Mediterranean Institute for Agriculture, Environment and Development & CHANGE—Global Change and Sustainability Institute, School of Science and Technology, Department of Biology, University of Évora, Pólo da Mitra, Ap. 94, 7006-554 Évora, Portugal

**Keywords:** respiration, alternative oxidase (AOX), calorespirometry, reactive oxygen species (ROS), oxidative stress, seed viability

## Abstract

**Simple Summary:**

Germination is a complex process that begins with rapid water uptake and a marked increase in oxygen consumption rate from initiation of aerobic respiration. The mitochondrial electron transport chain (ETC) in plants is a critical cellular respiratory system that has two different pathways: the cytochrome oxidase (COX) pathway and the alternative oxidase (AOX) pathway. The AOX enzyme plays a crucial role in energy production as well as in stress response. An AOX gene family study in *Pisum sativum* revealed three *AOX* genes (*PsAOX*). Transcript accumulation analysis revealed the involvement of all three *PsAOX* members in pea seed germination, albeit with high genotype variation. Calorespirometry was used to monitor metabolic changes during a germination trial using inhibitors of both respiratory pathways and proved to be useful for distinguishing differing respiratory activity in different cultivars. This study offers valuable insights into the *AOX* family composition in *P. sativum*, as well as their role during seed germination.

**Abstract:**

The alternative oxidase (AOX) is a ubiquinol oxidase with a crucial role in the mitochondrial alternative respiratory pathway, which is associated with various processes in plants. In this study, the activity of AOX in pea seed germination was determined in two pea cultivars, ‘Maravilha d’América’ (MA) and ‘Torta de Quebrar’ (TQ), during a germination trial using cytochrome oxidase (COX) and AOX inhibitors [rotenone (RT) and salicylic hydroxamic acid (SHAM), respectively]. Calorespirometry was used to assess respiratory changes during germination. In both cultivars, SHAM had a greater inhibitory effect on germination than RT, demonstrating the involvement of AOX in germination. Although calorespirometry did not provide direct information on the involvement of the alternative pathway in seed germination, this methodology was valuable for distinguishing cultivars. To gain deeper insights into the role of AOX in seed germination, the AOX gene family was characterized, and the gene expression pattern was evaluated. Three *PsAOX* members were identified—*PsAOX1*, *PsAOX2a* and *PsAOX2b*—and their expression revealed a marked genotype effect. This study emphasizes the importance of AOX in seed germination, contributing to the understanding of the role of the alternative respiratory pathway in plants.

## 1. Introduction

Legumes are used worldwide as the basis for a healthy diet, providing the most prominent source of protein [1]. Among the cultured legumes, pea (*Pisum sativum* L.) is one of the most widely spread crops in Europe, playing a very important role in human nutrition [2]. During their life cycle, plants are exposed to numerous environmental constraints with a negative impact on their development. From an agronomical perspective, this negative impact represents a decrease in yield and/or seed quality. The quality and viability of pea seeds are therefore agronomical traits with a high impact on both nutritional efficiency and sustainable crop production [3].

Seed viability is the capacity of a seed to germinate under suitable conditions. Seed germination starts with dry seed imbibition of water. Overall, the process of germination can be categorized into three distinct phases: Phase I, passive, is characterized by a rapid water uptake; Phase II is characterized by the initiation of metabolic activity, including the utilization of stored substrates and protein synthesis; and Phase III that marks the emergence of the radicle [4]. During imbibition, metabolic processes are activated to allow embryo germination and penetration of the radicle into the tissues surrounded by the seed coat. Activation of cellular respiration ensures the energy necessary for successful germination [5]. The rate of oxygen uptake by the seed increases during imbibition. Oxygen uptake in the re-hydrated seed causes accumulation of reactive oxygen species (ROS), which are also produced in response to biotic or abiotic stresses, functioning as signaling molecules [6,7]. ROS-scavenging mechanisms, involving antioxidant enzymes and antioxidant phenolic compounds, have a crucial role in controlling cellular ROS and consequently plant plasticity [7,8,9]. Plant plasticity, proposed by [10] as an agronomical trait, is nowadays considered in breeding programs for selection of more resilient genotypes to apply in organic agriculture [11]. Some genotypes have developed various strategies to minimize cellular damage and more efficiently resist/adapt to environmental constraints [12]. Plant plasticity depends on plant capacity to efficiently change the cellular program by activating a core set of multistress-responsive genes which control biochemical and physiological responses [13].

Mitochondrial signal perception and consequent canalization, as the central role in plant homeostasis, is now largely accepted [14,15]. The cyanide-insensitive alternative respiration pathway is localized in mitochondria, and the involvement of the alternative oxidase (AOX), an inner mitochondrial membrane enzyme, in maintaining metabolic homeostasis and signaling dynamics during plant growth during changing environmental conditions has been highlighted (reviewed in [16]). Velada and colleagues reported an increased *AOX1* transcript expression in cold-treated *Hypericum perforatum* L. seeds up to 24 h post incubation, and Campos and co-workers demonstrated a general increase in *AOX2a* expression under cold treatment for different carrot cultivars [17,18,19].

Considering the involvement of AOX in plant response to biotic/abiotic stresses, genes belonging to the AOX gene family have been proposed as functional markers for plant plasticity [10]. The involvement of AOX in seed germination was first proposed for chick pea (*Cicer arietinum* L.) [20] and cocklebur (*Xanthium* L.) seeds [21], considering that the alternative respiratory pathway is triggered by seed imbibition. In mung bean (*Vigna radiate* L.), Chaudhuri and Kar [22] demonstrated that germination is inhibited when seeds are treated with propyl gallate, an AOX inhibitor. Application of the AOX inhibitor SHAM (salicylic hydroxamic acid) inhibited the anaerobic germination and growth of deepwater rice (*Oryza sativa* L.) [23]. Furthermore, Fang and co-workers [24] proposed a model for H_2_S, a multifunctional gasotransmitter with pivotal functions in plant growth and development. In *Arabidopsis thaliana*, it promotes seed germination through the enhancement of AOX-mediated cyanide-resistant respiration. Notably, the endogenous production of H_2_S might increase during imbibition, consequently inducing an up-regulation in both *AOX1a* transcription and AOX protein abundance throughout seed germination. H_2_S operates as a mediator in the post-translational modification of AOX, maintaining it in an active and reduced state, thereby amplifying the rate of alternative respiration. Considering that seed germination involves the activation of several metabolic pathways, including respiration to provide the required energy for embryo development, the monitoring of parameters on seed respiration may allow the selection of more viable seeds. Phenotyping seeds to assess respiratory changes associated with cell reprogramming events was previously explored via calorespirometry [11,25,26]. This technique, which simultaneously measures heat and CO_2_ rates, previously used to explore phenotype pea seed viability [11], has been used as a screening tool to assess respiratory changes in a variety of plant species and biological processes [25,27]. Recently, calorespirometry has emerged as a valuable non-destructive phenotyping technique for the identification of genotypes that exhibit enhanced germination resilience to temperature fluctuations, as well as for assessing seed vigor in pea [11].

In plant breeding, the plasticity to adapt to different environmental conditions is a desirable trait (first proposed by [10]). The link between AOX and respiratory parameters was previously established by Campos and co-authors [25]. Thus, it is essential to explore the link between AOX and respiratory parameters during germination. The aim of this work was to investigate the involvement of AOX in pea seed germination by using inhibitors of the cytochrome and alternative respiratory pathways. Calorespirometry was used to accesses cellular respiratory changes. AOX gene/protein expression analysis was accessed during germination, and ROS levels were determined to establish a link between AOX gene/protein pattern change and the establishment of cellular ROS balance.

## 2. Materials and Methods

Seeds of *P. sativum* L. cv. ‘Maravilha d’América’ and cv. ‘Torta de Quebrar’, obtained from Flora Lusitana Lda. (Cantanhede, Coimbra, Portugal), were used in the experiments.

Three experiments were conducted: (1) a germination trial with respiratory inhibitors; (2) an experiment to evaluate changes in respiratory metabolism (cyanide sensitive and insensitive pathways) at early germination stages; and (3) an experiment to verify the involvement of AOX in seed germination (at transcript and protein expression level) and in the establishment of ROS homeostasis.

### 2.1. Involvement of the Cyanide-Insensitive Respiratory Pathway in Seed Germination

Seeds of each pea cultivar (cv. ‘Torta de Quebrar’ and cv. ‘Maravilha d’América’) were placed in a 3 cm diameter-well tray and covered with cotton soaked with the two different respiratory inhibitor solutions. Rotenone (RT, 5 µM) is a botanical pesticide used to investigate the involvement of the cyanide-sensitive cytochrome pathway. Rotenone inhibits complex I of the electron transport chain [28], and results in the inability to produce ATP since electrons from NADH do not enter in the ETC [29]. SHAM (10 mM) was used to inhibit AOX activity, allowing for investigation of the involvement of the cyanide-insensitive alternative respiratory pathway. SHAM inhibits AOX by blocking the largely uninhibited flow of electrons [30]. Another set of pea seeds were soaked with sterilized tap water and used as control samples (CT). Seeds of the three treatments (RT, SHAM, and CT) were kept permanently wet during the experiment and were incubated in complete darkness under 75% relative humidity and 25 °C temperature, one of the temperatures used in a previous study of the same pea cultivars [11]. A total of 240 seeds per condition and cultivar were considered (24 replicates of 10 seeds each).

To calculate germination rate, seeds were observed daily over the six-day experiment. A seed was considered germinated when a discernible radicle extended beyond 1 mm [31]. Seed germination achieved over six days (cumulative germination) was calculated using the equation $Germination\;\left(\%\right)=(Number\;of\;germinated\;seeds/TS)\times 100$, where *TS* corresponds to the total number of seeds used to establish the experiment.

### 2.2. Evaluation of Respiratory Changes during Germination

The involvement of the cytochrome and alternative respiratory pathways in seed respiration was evaluated via calorespirometry using seeds of the two pea cultivars (cv. ‘Torta de Quebrar’ and cv. ‘Maravilha d’América’) soaked in RT (5 µM), SHAM (10 mM), or water (CT). Calorespirometric measurements were made following the procedure previously optimized [11], and performed after 16 h of seed imbibition, the period required to reach the required stability in calorespirometric parameters, coincident to the complete seed water uptake. A total of 10 replicates (single seeds) were considered per treatment and cultivar.

Calorespirometric measurements run in isothermal mode at 25 °C, a temperature selected based on previous results [11]. The measured heat rates allowed for the calculation of specific heat rates (R_q_) and specific CO_2_ production rates (R_CO_2__) [32]. The values of R_q_ and R_CO_2__ were used to calculate the structural biomass formation rate (R_struct _bio_) and carbon use efficiency (Ɛ) [32]. Values of R_q_, R_CO_2__, and R_struct_bio_ were normalized to seed dry mass before imbibition. Ɛ is a unitless fraction and therefore does not require normalization.

### 2.3. Analysis of P. sativum AOX Gene and Protein Expression

To complement the investigation of the involvement of AOX in seed germination, transcript abundance of *AOX* pea genes (*PsAOX1*, *PsAOX2a*, and *PsAOX2b*) and AOX protein expression were quantified in CT- and RT-treated seeds of the two-pea cultivar (selected based on the contrasting behavior according to the previous experiments (see details in [11])).

#### 2.3.1. Identification and Characterization of *P. sativum AOX* Gene Members

Since the *AOX* gene sequences of *P. sativum* were not publicly available, prior to the expression analysis, the sequences and gene structure of the *AOX* gene family members needed to be identified in this plant species. For this, all pea sequences homologous to *AOX Glycine max* sequences were retrieved, and a BLAST search was performed at the pea genome database (available at https://urgi.versailles.inra.fr/Species/Pisum/Pea-Genome-project, accessed on 3 June 2021). *AOX* sequences retrieved from *G. max* were used as queries because it belongs to the same family Fabaceae. Sequences identified in the *P. sativum* database were further used as secondary queries. To verify the homology with *AOX* of the sequences identified at the pea genome database, a Blastn analysis at the NCBI (National Center for Biotechnology Information, https://www.ncbi.nlm.nih.gov/, accessed on 3 June 2021) was conducted.

For *PsAOX* gene structure analysis, the software Splign (https://www.ncbi.nlm.nih.gov/sutils/splign/splign.cgi?textpage=online&level=form, accessed on 5 July 2023) was used. To compare *PsAOX* gene structure with other *AOX* genes from other plant species, genomic and transcript sequences of five additional species belonging to the Fabaceae family (*Glycine max*, *Medicago trucatula*, *Phaseolus vulgaris*, *Trifolium pratense*, and *Vigna unguiculata*) were retrieved from EnsemblPlants databases (https://plants.ensembl.org/index.html, accessed on 3 June 2021). The GSDS 2.0 software (available at http://gsds.gao-lab.org/, accessed on 21 July 2023) was used to draw the scheme with the exon/intron composition.

To get the correct classification of *P. sativum AOX* genes (*PsAOX*), protein sequences were aligned with *Glycine max* AOX sequences, and the classification adopted was based on [33]. CLC Genomics Workbench 11.0.1 software (ClCbio, Aarhus N, Denmark) was used to edit AOX sequences and perform alignment.

Protein subcellular localization and position of the cleavage sites of mitochondrial targeting signals were predicted by using the translated peptide corresponding to exon 1 in the TargetP software [34] (freely available at http://www.cbs.dtu.dk/services/TargetP/, accessed on 4 July 2023). The prediction of putative isoelectric point (pI) and the molecular weight was conducted by using the PeptideMass tool, freely available at Expasy software (http://web.expasy.org/peptide_mass/, accessed on 4 July 2023).

To better evaluate the relation between the identified sequences, a phylogenetic relationship study was conducted using the deduced peptide sequences of the pea genes and genes from Liliopsid (14 species) and Magnoliopsid (38 species) species retrieved from Phytozome (https://phytozome.jgi.doe.gov/pz/portal.html, accessed on 3 June 2021) (details of sequences are in Supplementary Materials Tables S1 and S2). Retrieved sequences were aligned in MUSCLE (http://www.ebi.ac.uk/Tools/msa/muscle/, accessed on 15 June 2021), following the default settings to generate an output Pearson/FASTA file. MEGA 7 [35] was used to construct a phylogenetic tree using the neighbor-joining (NJ) method [36] with bootstrap analysis using 1000 replicates, “number of differences” as the substitution model and “pairwise deletion” for gaps/missing data treatment. For a graphical view, the tree was edited in Fig Tree v14.0 software ([37], Edinburgh, UK) (http://tree.bio.ed.ac.uk/software/figtree/, accessed on 16 June 2023).

#### 2.3.2. RNA Extraction, cDNA Synthesis, and Gene Expression Analysis

The analysis of *PsAOX* gene expression was conducted in both cultivars (cv. ‘Torta de Quebrar’ and cv. ‘Maravilha d’América’) considering two treatments: control (CT) and rotenone (RT). Samples were collected at five different time-points: 0 h (previous to imbibition), 4, 8, 12, and 16 h during imbibition (hpi). Each sample consisted of a pool of four seeds. Four biological replicates were considered per timepoint and treatment (CT and RT). Samples were homogenized with liquid nitrogen, crushed into a fine powder, and stored at −80 °C until further processing.

RNA isolation was performed using a Maxwell^®^ 16 LEV simplyRNA Cells Kit from Promega (Madison, WI, USA) on a Maxwell^®^ 16 Instrument (Promega, Madison, WI, USA) following the manufacturer’s protocol. Total RNA was eluted in 50 μL sterile Nuclease-free water. RNA concentration was further determined using a Thermo Scientific^TM^ Nanodrop^TM^ 2000C spectrophotometer from Thermo Scientific (Wilmington, DE, USA). Both 260/280 and 260/230 ratios were used to assess RNA purity.

RNA integrity was analyzed in a 2% denaturation agarose gel electrophoresis loaded with 4 µL of total RNA and 2 μL of RNA Gel Loading Dye (Thermo Scientific). Electrophoresis was run at a constant voltage of 100 V for 60 min. After electrophoresis, gel was visualized using a Gene Flash Bio Imaging system (Syngene, Cambridge, UK) after staining it with GreenSafe solution (NZYtech, Lisbon, Portugal). One μg of total RNA was used for cDNA synthesis using the SensiFAST™ cDNA Synthesis Kit (Bioline, Luckenwalde, Germany) following the supplier’s instructions. Briefly, 4 µL of TransAmp Buffer (5x) and 1 µL of reverse transcriptase (RT) were added to the RNA of each sample. The cDNA synthesis was conducted in a 20 µL total volume reaction following a program consisting of 10 min at 25 °C, followed by 15 min at 42 °C and 15 min at 48 °C. For inactivation of the enzyme, a 5 min step at 85 °C was conducted. cDNA samples were further diluted 1:5 and stored in aliquots at −20 °C until further analysis.

Gene expression analysis was performed via reverse transcription quantitative PCR (RT-qPCR) in an Applied Biosystems 7500 Real-Time PCR System (Applied Biosystems, Foster City, CA, USA) in 96-well plates. Reactions were conducted with 2 µL of previously diluted cDNA, 1x of SensiFASTTM SYBR^®^ Lo-ROX Master Mix (Bioline, Luckenwalde, Germany), and 300/400 nM of specific primers for target and reference genes (Table S3) in a total volume of 16 µL. Primers for *PsAOX* members (*PsAOX1*, *PsAOX2a*, *PsAOX2d*) were designed using Primer3Plus software (https://www.bioinformatics.nl/cgibin/primer3plus/primer3plus.cgi, accessed on 17 April 2021) (Table S3). The genes encoding for Protein POB (*PsPOB*) and GTP-binding protein Sar1 (*PsSAR1*) were previously selected as the most stable genes and are here used as reference genes (Table S3).

Amplification was performed according to the details shown in Table S3. The PCR efficiency (*E*) was calculated via the equation *E* (%) = (10^(−1/slope)^−1) × 100 [19] through a standard curve of a 4-fold dilution series. The specificity of primers was evaluated via a melting curve analysis and further observation of a single peak. Contaminations and formation of primer secondary structures were evaluated by running no-template control (NTCs) samples for each primer pair. Gene expression analysis was run for four biological replicates and two technical replicates of each biological.

To obtain the normalized gene expression values, the procedure described by [19] was followed. Briefly, the normalization factor was first calculated as the geometric mean of the relative quantities (RQ) of both reference genes. Target gene expression corresponding to each sample was further calculated as the ratio between the RQ of the target gene and the RQ of the corresponding normalization factor.

#### 2.3.3. Analysis of *P. sativum* AOX Protein Expression by Western-Blot Analysis

##### Protein Extraction and Quantification

Samples collected from cv. ‘Torta de Quebrar’ and cv. ‘Maravilha d´América’ at 0, 4, and 16 hpi were used for PsAOX expression analysis. Four biological replicates, each one consisting of a pooled sample of four seeds, were considered per timepoint and cultivar. A total of 50 mg of previously homogenized seeds were placed in 2 mL polypropylene tubes to further proceed with protein extraction following the method of Mamontova and coworkers [38] with some modifications previously optimized [11]. Concisely, cold (4 °C) phenol extraction buffer [0.7 M sucrose, 0.1 M KCl, 5 mM ethylenediaminetetraacetic acid (EDTA), 20 mM Dithiothreitol (DTT), 1 mmol/L phenylmethylsulfonyl fluoride (PMSF), and 0.5 M tris-HCl buffer (pH 7.5)] was added to the plant material. After being vigorously mixed for 30 s, cold phenol (4 °C) saturated with 0.5 mol/L tris-HCl buffer (pH 7.5) was added to the samples. These samples were further incubated for 30 min at 900 rpm and 4 °C, followed by centrifugation (15,000× *g*, 30 min, 4 °C). Subsequently, the phenolic (upper) phase was transferred to a new tube and proteins were precipitated by adding ice-cold 0.1 mol/L ammonium acetate in methanol. After overnight incubation at −20 °C, the resulting pellet (protein fraction) was collected via centrifugation (10 min, 5000× *g*, 4 °C) and washed twice with two volumes of methanol (relative to the volume of the phenol phase) and twice with the same volume of acetone (both at 4 °C). Each time, the samples were centrifuged (5000× g, 10 min, 4 °C) after resuspension. Finally, the purified pellets were air-dried in a fume hood for 1 h and then reconstituted in 1 mL of solubilization buffer (7 M urea, 2 M thiourea, 4% CHAPS, 100 mM tris-HCl, pH 7.5). Total protein quantification was carried out using the Pierce 660 nm Protein Assay Reagent (Thermo Scientific™).

##### Western Blotting

A total of 25 µg of protein from each sample was run per lane in a 14% polyacrylamide SDS-PAGE electrophoresis mini-gel (Protean xi, Bio-Rad Laboratories, Lisbon, Portugal) using Laemmli buffer [39]. Each sample was run in triplicate. Four biological replicates of each timepoint and cultivar were used. Electrophoresis was run at constant voltage (130 V) until the dye front reached the end of the gel.

After protein separation via SDS-PAGE electrophoresis, proteins were transferred to a PDVF membrane via electroblotting using a Semi-Dry Trans-Blot Turbo Transfer (Bio-Rad) system. After transferring, blocking was performed with 5% non-fat dry milk in TBS-Tween 20 for 2 h, with agitation, at room temperature. Before incubation with primary antibodies, the membrane was cut, and the upper part was incubated with primary antibody anti-actin (Agrisera, Vännäs, Sweden, AS13 2640; dilution: 1:1000), and the lower part with primary antibody anti-AOX1/2 (Agrisera AS04054; dilution: 1:1000). PsActin was used as endogenous control for the normalization of data achieved for PsAOX. The incubation with primary antibodies was performed overnight at 4 °C. Bands of PsAOX and PsActin were detected with an alkaline phosphatase-linked secondary antibody (anti-rabbit, Agrisera AS09607, 1:10,000 dilution), using a chemifluorescent substrate (ECF Plus Western Blotting Detection Reagents, GE Healthcare, Lisbon, Portugal). Membranes were analyzed in a Gel-Doc XR Imaging system (Bio-Rad). To achieve semi-quantitative protein expression values, the Bio-Rad Image Lab 5.2.1 (Bio-Rad) software was used. Optical density was obtained via densitometric analysis. The values are background corrected, which was applied to each band by normalizing an adjacent region of the membrane, and control protein correction was conducted.

### 2.4. Total ROS Determination

Plant material collected from cv. ‘Torta de Quebrar’ and cv. ‘Maravilha d´América’ at 0, 4, 8, 12, and 16 hpi were used for total ROS determination. Four biological replicates, each one consisting of a pooled sample obtained from four seeds, were considered per timepoint and cultivar. The fluorogenic dye 2′,7′-dichlorofluorescein diacetate (DCFDA) was used to quantify the levels of ROS released from dry and imbibed seeds, according to Jambunathan [40] with some modifications. Briefly, 100 mg plant material taken from each sample, previously homogenized with liquid nitrogen, was transferred to a 2 mL polypropylene tube containing 1 mL of 10 mM Tris–HCl, pH 7.2. After mixing, the samples were centrifuged at 12,000× *g* for 20 min at 4 °C. Afterwards, the supernatant was transferred to a new tube and 100 μL of supernatant diluted with 900 μL of 10 mM Tris–HCl (pH 7.2). To these diluted samples, 10 μL of 1 mM DCFDA was added (final concentration is 10 μM), followed by vortexing and incubation in dark for 10 min. Subsequently, a volume of 25 μL was pipetted into a 24-well plate, and the emitted fluorescence was measured in a PikoReal^®^ 24 (Thermo Scientific) thermocycler (setting the program for two cycles of 15 s at 25 °C), with maximum excitation and emission spectra of 495 nm and 529 nm, respectively. Total ROS content was expressed in relative fluorescence units (RFU).

### 2.5. Statistical Analysis

Statistical analyses were performed using SPSS program version 22.0. Normality and variance homogeneity were checked in all cases, and when required, the data were log transformed.

In the case of germination, data did not fulfil the conditions for a parametric analysis; therefore, they were analyzed by Kruskal–Wallis tests followed by a Dunn *post hoc* test.

Calorespirometry data were analyzed by a two-way ANOVA with cultivar and inhibitor treatment considered as main factors. When the interaction between the main factors was found to be non-significant, the main effects were examined separately by a *t*-test. *PsAOX* gene expression, PsAOX protein expression, and ROS concentration data were analyzed via a three-way ANOVA where cultivar (‘Maravilha d’América’ and ‘Torta de Quebrar’), treatment (CT and RT), and imbibition period (0, 4, 8, 12, and 16 hpi in the case of gene expression and ROS concentration; 0, 4, and 16 hpi in the case of protein expression) were considered as main factors (see details of experimental design in Figure S1). Since significant interactions were found between those main factors in *PsAOX* gene and protein expression data, the analyses were split for each cultivar. Subsequently, a two-way ANOVA was conducted in each case to study the effects of the inhibitor treatment and imbibition time, as well as their interaction. When the interaction between the main factors was not significant, the main effects were examined separately via a Duncan post hoc test (or, alternatively, via a Dunn test, when data did not fulfill normality or variance homogeneity conditions). When the interaction between both factors was significant, a multiple comparison test was conducted to compare the means of all treatment/imbibition time combinations (Duncan test or Dunn test when data did not fulfill normality or variance homogeneity conditions).

## 3. Results

### 3.1. Involvement of Respiratory Pathways in Seed Germination

The germination rate was evaluated on the cvs. ‘Torta de Quebrar’ and ‘Maravilha d’América’ in seeds imbibed in SHAM, RT, or water (CT) (results shown in Table 1).

A similar pattern was observed across both cultivars, as the percentage of germination tended to decrease when inhibitors were used. In cv. ‘Torta de Quebrar’, a significant decrease in germination was observed when the principal respiration path was inhibited with RT (Cyt pathway), and an even higher decrease occurred when SHAM was applied. However, in cv. ‘Maravilha d’América’, this decrease was less pronounced, and not statistically significant when compared the CT- with the RT-treated seeds. Nonetheless, significant differences were observed when SHAM was applied, as compared to the CT treatment.

When the cultivars were compared, cv. ‘Torta de Quebrar’ tended to have higher germination rates than cv. ‘Maravilha d’América’ for both CT- and RT-treated seeds, although not statistically significant. Moreover, when alternative respiration was inhibited by treating the seeds with SHAM, germination was highly inhibited, and a very low germination rate was seen, with lower values observed in the cv. ‘Torta de Quebrar’ (5%).

### 3.2. Evaluation of Metabolic and Respiratory Changes during Seed Germination

**Figure 1:**
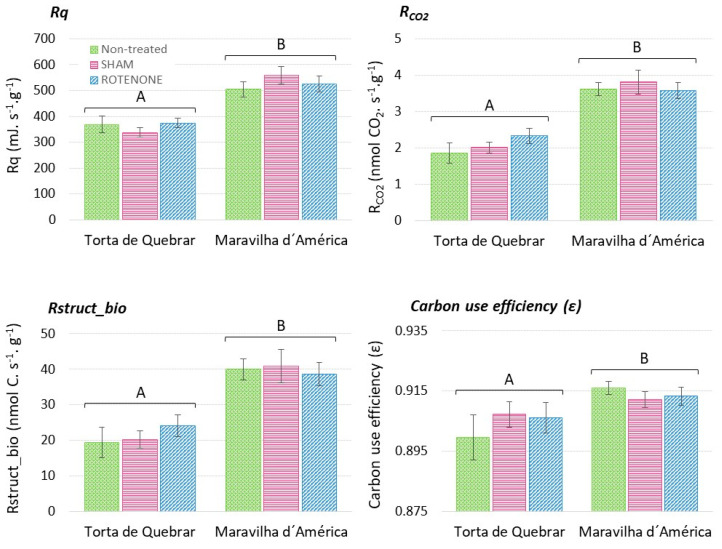
Calorespirometric parameters for two pea (*Pisum sativum* L.) cultivars (cv. ‘Torta de Quebrar’ and cv. ‘Maravilha d’América’) measured at 25 °C after 16 h imbibition in a solution with SHAM (salicylic hydroxamic acid), Rotenone (RT), or without inhibitors (CT). R_q_: respiratory heat rate; R_CO_2__: CO_2_ production rate; R_struct_bio_: rate of growth of structural biomass; Carbon use efficiency (ε): substrate carbon conversion efficiency. Bars indicate the mean value of six measurements ± standard error. Different capital letters indicate significant differences between cultivars, according to a *t*-test. Statistical significance was considered for *p* ≤ 0.05.

The two-way ANOVA test conducted to evaluate the effect of inhibitor treatments and the effect of the cultivar on calorespirometric parameters did not show a significant effect of SHAM and RT on R_q_, R_CO_2__, R_struct_bio_, or ε (Figure 1, Table 2). Only the cultivar had a significant effect on the calorespirometric parameters, which were significantly higher in cv. ‘Maravilha d’América’ germinating seeds than in ‘Torta de Quebrar’ (Figure 1, Table 2).

### 3.3. Quantification of AOX Transcript Level during Germination in Seeds Imbibed with COX-Pathway Inhibitor

#### 3.3.1. Identification and Characterization of *P. sativum AOX* Gene Members

Three sequences were identified at the genome databases, the Psat2g142680.1, Psat2g058520.1, and Psat7g098200.1, located on different chromosomes, namely, chr. 6 (LG2), chr. 2 (LG1), and chr. 7 (LG7), respectively.

Gene structure analysis revealed a four-exon structure in both Psat2g058520.1 and Psat7g098200.1, with the three last exons exhibiting conservation on size (exon 2: 129, exon 3: 489, and exon 4: 57 bp), interrupted by three highly variably sized introns (Figure S2). The Psat2g142680.1 revealed a three-exon structure due to an event of intron loss by fusion of exons 2 and 3. The high variability of intron size is the main factor contributing to gene size diversity, Psat7g098200.1 with 3790 bp (999 bp transcript size, 332 amino acid residues encoded), Psat2g058520.1 with 1417 bp (969 bp transcript size, 322 amino acid residues encoded), and Psat2g142680.1 with 1277 bp (894 bp transcript size, 297 amino acid residues encoded).

**Figure 2:**
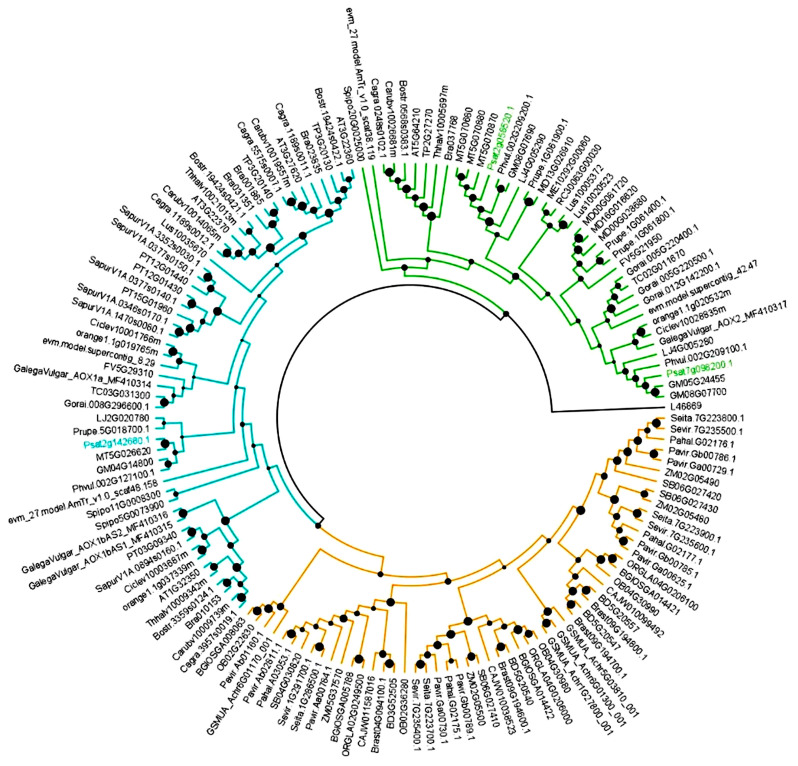
Neighbor-joining (NJ) tree showing the phylogenetic relationships among deduced *Pisum sativum* AOX sequences and AOX sequences retrieved from 38 Magnoliopsid and 14 Liliopsid plant species. PsAOX1 sequence is highlighted in blue and PsAOX2-subfamily members are highlighted in green. The analysis encompassed 181 putative AOX sequences from higher plants (details regarding accession numbers and corresponding plant species can be found in Supplementary Materials Tables S2 and S3). The construction of the neighbor-joining (NJ) tree employed the entire peptide sequences. Alignment was bootstrapped with 1000 replicates using the NJ method through MEGA 7 software. The scale bar on the diagram denotes the relative amount of change along the branches. AOX proteins can be divided into three main branches: the yellow corresponds to the Liliopsid AOX1 members; the blue corresponds to the AOX1 Magnoliopsid members; and the green corresponds to the AOX2-subfamily members from Magnoliopsid members.

To classify *P. sativum* genes and their corresponding proteins, a phylogenetic analysis was conducted using protein sequences, which resulted in the categorization of the sequences into the clades AOX1 and AOX2. The dendrogram revealed three main clusters, one grouping all sequences of Liliopsid plant species (in yellow in Figure 2), and other two grouping the sequences of Magnoliopsid species. Sequences of Magnoliopsid are grouped into two different clusters based on whether they belong to AOX1 (in blue in Figure 2) or AOX2-subfamily (in green in Figure 2). A single pea AOX member clustered on the AOX1-subfamily (Psat2g142680.1), and two members clustered on the AOX2-subfamily (Psat7g098200.1 and Psat2g058520.1).

**Figure 3:**
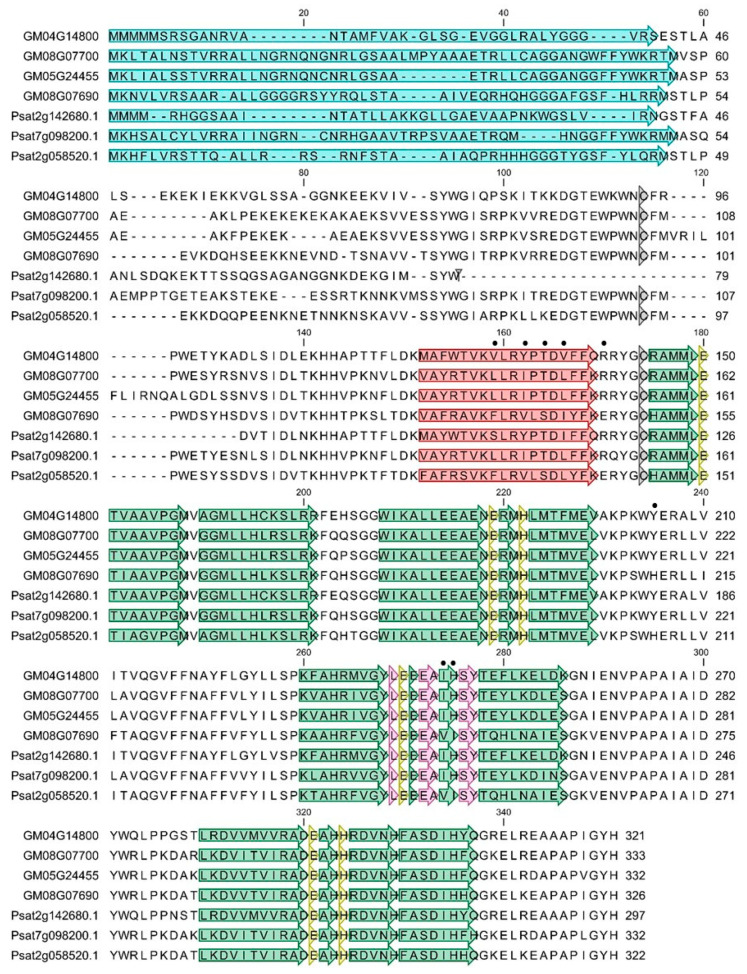
Multiple alignment of putative amino acid translated sequences of previously reported AOX proteins from G. max (GM04G14800, GM08G07690, GM08G07700, and GM05G24455) and AOX from *Pisum sativum* (PsAOX1—Psat2g142680.1; PsAOX2a—Psat7g098200.1; and PsAOX2d—Psat2g058520.1). Alignment was conducted utilizing the CLC Genomics Workbench 11.0.1 software. The data were achieved from the publicly accessible Plaza v2.5 web-based database, available at http://bioinformatics.psb.ugent.be/plaza/versions/plaza_v2_5/, accessed on 3 June 2021. Amino acid variations are highlighted in red, while deletions are denoted by minus signs. Blue boxes highlight the putative mitochondrial transit peptides (mTP). Regions containing the two conserved cysteins (CysI and CysII), pivotal for AOX protein dimerization through S–S bond formation, are marked by dark grey boxes. Helices α1 and α4, which comprise the hydrophobic domain on the AOX molecular surface and contribute to membrane attachment, are depicted in red. Helices α2, α3, α5, and α6, which constitute the four-helix bundle accommodating the diiron center, are presented in green [38]. Amino acid residues coordinating the diiron center (E, glutamate; and H, histidine), as well as those interacting with the inhibitor, are delineated within yellow and light pink boxes, respectively. Residues that allow for distinguishing the AOX2d members are indicated with filled black circles (according with [33]).

The results of the phylogenetic analysis are supported by the specific AOX motifs identified by a multiple-sequence alignment including AOX-translated peptides of *P. sativum* (PsAOX) and *G. max* (Figure 3). The three PsAOX-deduced sequences, encoded by the three identified transcripts, revealed structural features usually found in AOX of most of the higher plants. The two conserved cysteines (CystI and CystII) were identified in both PsAOX2-subfamily members, while the PsAOX1 lacks CystI. Di-iron-binding sites were identified in all PsAOX members. All *P. sativum* AOX-translated peptides were predicted to be translocated to mitochondria after processing (mTP score of 0.70, 0.92 and 0.98 for PsAOX1, PsAOX2a and PsAOX2d, respectively). Even with sequence diversity at the N-terminal region, few implications were detected at the mitochondrial signal transit peptide, which changed between 41 and 51 amino acid residues (STP seen in blue at Figure 3).

#### 3.3.2. Analysis of *PsAOX* Expression Pattern

To verify whether the expression levels of *PsAOX* genes changed during 16 h pea seed germination, and to study how the inhibition of the cyanide-sensitive respiratory pathway affects the alternative pathway, a RT-qPCR analysis was performed.

**Figure 4:**
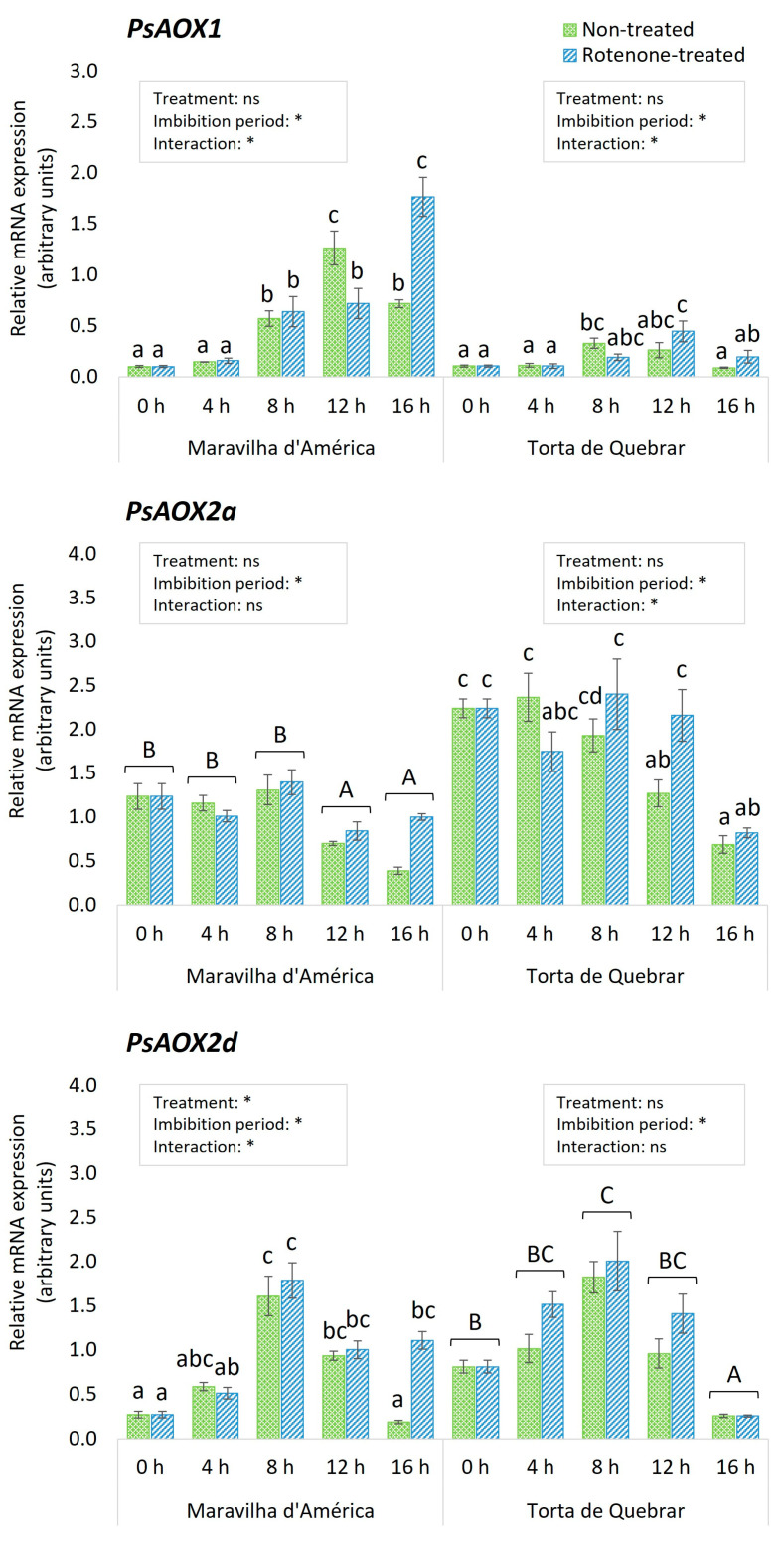
Relative expression of *PsAOX1*, *PsAOX2a*, and *PsAOX2d* genes during germination of *Pisum sativum* seeds (cv. ‘Maravilha d’América’ and cv. ‘Torta de Quebrar’) during 16 h of imbibition in water (non-treated) or in rotenone (rotenone-treated). The relative expression values achieved at each time point are depicted as the mean ± standard error of four biological replicates. The square above each cultivar indicates the results of the two-way ANOVA for the effects of the inhibitor treatment and imbibition time. Statistical significance at *p* ≤ 0.05 is indicated with an asterisk, and “ns” stands for non-significant differences. When the interaction between the main factors was not significant, different capital letters indicate significant differences among imbibition times (0, 4, 8, 12, and 16 hpi). When the interaction was significant, different low-case letters represent significant differences among all treatment/imbibition time combinations, according to Duncan or Dunn test (when data did not follow a normal distribution).

The results of the three-way ANOVA test conducted to evaluate the effects of inhibitors, cultivars, and different imbibition periods on *PsAOX* gene expression are given in Figure 4 and Table 3. For all genes, there was a significant interaction between the imbibition period and the cultivar, and there was an interaction between inhibitor treatment and imbibition period for *PsAOX1* and *PsAOX2a* expression. Additionally, a significant interaction was found among the three factors for *PsAOX1* expression.

Due to the interactions identified by the three-way ANOVA, a separate statistical analysis was conducted for each cultivar, where the effects of the treatment and the imbibition time were studied along with their interaction by a two-way ANOVA.

The cvs. ‘Maravilha d’América’ and ‘Torta de Quebrar’ presented very different *PsAOX1*, *PsAOX2a*, and *PsAOX2d* expression patterns.

A significant interaction was found between the two factors in both cultivars. In cv. ‘Maravilha d’América’, in the case of non-treated seeds (imbibed in water, CT), the expression increased until reaching its peak at 12 hpi and subsequently decreased, while in RT-treated seeds, the expression showed a progressive increase, continuing until 16 hpi. Differences between CT and RT treatments were found at 12 hpi, when CT seeds showed higher *PsAOX1* expression (12-fold change in comparison with 0 hpi, *p* ≤ 0.005), and at 16 hpi, when RT-treated seeds had higher expression levels (17-fold change in comparison with 0 h, *p* ≤ 0.005) (Figure 4).

Expression of *PsAOX1* was generally lower in cv. ‘Torta de Quebrar’. In CT seeds, the expression increased over the time and reached a plateau between 8 and 12 hpi, decreasing subsequently to the initial levels. In RT-treated seeds, a peak of expression was observed at 12 hpi. No significant differences were found between CT- and RT-treated seeds at any time point (Figure 4).

The expression pattern of *PsAOX2a* was very different than that of *PsAOX1*. Overall, expression levels were higher in both cultivars. In cv. ‘Maravilha d’América’, the imbibition time had a significant effect on *PsAOX2a* expression. While it remained stable from 0 to 8 hpi, a significant decrease was observed at 12 and 16 hpi.

In cv. ‘Torta de Quebrar’, a significant interaction was found between the imbibition time and the treatment on *PsAOX2a* expression. In non-treated seeds, the expression level of this gene started to decrease after 8 hpi and reached the minimum value at 16 hpi. In RT-treated seeds, the decrease in gene expression was observed later, at 16 hpi. Significant differences between treatments were only observed at 12 hpi, and RT-treated seeds were the ones showing the higher expression levels. Comparing the transcript level of *PsAOX2a* between the two cultivars, significantly higher values were observed in cv. ‘Torta de Quebrar’, independent of the germination conditions (CT or RT) (Figure 4).

Concerning *PsAOX2d*, in cv. ‘Maravilha d’América’, a significant interaction was observed between the two factors (treatment and imbibition period). In both CT- and RT-treated seeds, the expression reached a peak at 8 hpi. However, while in CT seeds, the expression at 16 hpi decreased to the initial levels, in RT-treated seeds, the expression did not decrease further and remained the same as at 12 hpi. Significant differences in *PsAOX2d* expression between CT and RT seeds were observed only at 16 hpi. In this instance, the RT-treated seeds exhibited the highest gene expression levels.

In cv. ‘Torta de Quebrar’, the imbibition period had a significant effect on *PsAOX2d* gene expression, and the interaction between both factors was not significant. The expression of this gene increased progressively until reaching a peak at 8 hpi, and it further decreased to lower values than the initial ones at 16 hpi.

#### 3.3.3. Analysis of PsAOX by Immunoblotting (Western Blotting)

Concerning PsAOX protein expression, one band with an apparent molecular mass of 35 kDa was identified as AOX (Figure S3). In silico determination of molecular weight of the three PsAOX members revealed values of 33 kDa, 37 kDa, and 38 kDa for PsAOX1 (pI 7.82), PsAOX2d (pI 8.99), and PsAOX2a (pI 9.18), respectively.

**Figure 5:**
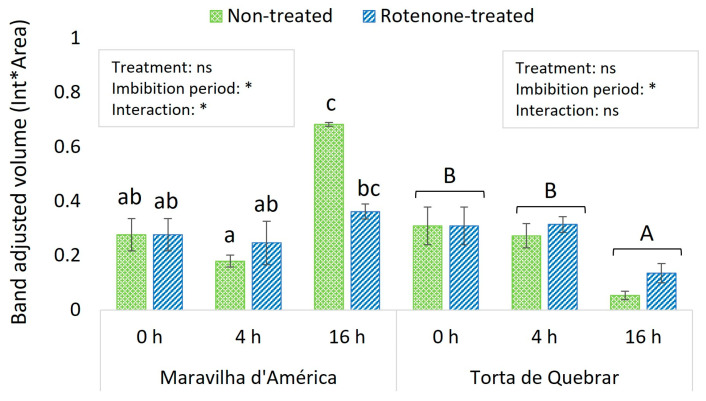
Expression of PsAOX proteins during germination of two *Pisum sativum* cultivars over 0, 4, and 16 h of seed imbibing in water (non-treated) or rotenone solution. Bars indicate the mean value of four replicates ± standard error. The box on the top of each cultivar indicates the results of the two-way ANOVA for studying the effects of the inhibitor treatment and imbibition time. Statistical significance at *p* ≤ 0.05 is indicated with an asterisk, and “ns” stands for non-significant differences. Different letters above the bars represent the significant differences according to the Duncan or Dunn test (when data did not follow a normal distribution).

Significant interactions were found between the cultivar and imbibition period, and between the cultivar, imbibition period, and treatment (Table 4). When the analysis was conducted separately for each cultivar, in cv. ‘Maravilha d’América’, a significant interaction was also found between imbibition period and treatment. In CT seeds, protein expression was maximum at 16 hpi, but in RT-treated seeds, PsAOX expression remained constant along the 16 hpi. Protein expression did not differ between CT and RT seeds at any time point (Figure 5).

In cv. ‘Torta de Quebrar’, no interaction was found, and the imbibition period had a significant effect in PsAOX expression. Both CT- and RT-treated seeds followed a similar pattern. The expression of the protein was stable from 0 to 4 hpi, but significantly decreased at 16 hpi (Figure 5).

#### 3.3.4. Quantification of ROS Levels during Germination

The three-way ANOVA conducted to study the effect of the inhibitor treatment, cultivar, and imbibition period on ROS concentration showed that both the cultivar and the period of imbibition had a significant effect, but not the treatment. No significant interactions were found between any of the factors, although the probabilities for imbibition time x treatment and imbibition time x cultivar were close to 0.05 (*p* = 0.089 and *p* = 0.065, respectively) (Table 5a).

The cv. ‘Torta de Quebrar’ had significatively higher ROS concentration than cv. ‘Maravilha d’América’, and overall, an ROS concentration increased from 0 to 8 hpi, slightly decreased at 12 hpi, and increased again at 16 hpi (Table 5b).

## 4. Discussion

Germination is a complex process, driven by a diversity of cellular processes, i.e., transcription, translation, cell elongation, cell cycle activation, repair mechanisms, and organelle reconstitution [6]. In this work, we first studied the involvement of AOX in controlling seed germination, which is commonly conducted through the use of SHAM as an AOX enzyme activity inhibitor [20,41,42]. The use of SHAM in the pea germination trial resulted in a significant decrease in germination, in contrast to the use of RT, which did not show a significant effect in case of cv. ‘Maravilha d’América’. The results demonstrate the importance of the alternative respiratory pathway in pea seed germination, and agrees with the results obtained in *Citrullus lanatus* and *Daucus carota* seed germination in which an inhibitory effect of SHAM on germination was also reported [26,43].

Although both COX and AOX pathways are involved at early stages of germination, it has been suggested that the AOX pathway plays the most critical role [26,44], probably due to the low affinity of AOX for O_2_ [45]. To confirm the hypothesis that the AOX pathway has a more crucial role in seed germination than COX, seed metabolism and respiration were monitored via calorespirometry using seeds soaked in a SHAM (inhibitor of AOX) or RT (inhibitor of cytochrome oxidase pathway) solution. The potential of calorespirometry as a phenotyping tool to predict growth potential and temperature response has been previously studied [26,30], but only recently it has been applied to phenotype seed viability, high plasticity upon temperature changes, and seed physiological quality [11]. Similar to the results obtained by [11], we also found that the cv. ‘Torta de Quebrar’ showed the lowest values of R_q_, R_CO_2__, and R_struct_bio_ independently of whether inhibitors were used or not.

In our study, we did not find any effect of SHAM or RT on respiration rate or R_struc_bio_ (i.e., anabolic rates or rate at which C is being incorporated into new tissues). In contrast, in a recent study, restriction of the COX and AOX pathways in a salt-tolerant cultivar caused a significant reduction in CO_2_ assimilation rates compared with a salt-sensitive cultivar [46]. Stressful conditions lead to changes in metabolic pathways, which are reflected in low values of ε [47]. A possible explanation for our results is that when an inhibitor is used, the other pathway compensates and provides the whole respiratory activity. In fact, AOX and COX compete for electrons, and when one pathway is inhibited, the other one can accept a greater number of electrons [48]. Therefore, due to the lack of differences in calorespirometric parameters, the method is not suitable for evaluation of the role of each respiratory pathway on germination, although it is clearly applicable for the discrimination of genotypes.

To demonstrate that AOX has a crucial role during seed germination, both transcript accumulation and protein expression with or without RT for inhibiting the COX pathway were studied. However, for this purpose, a characterization of the *P. sativum AOX* gene family was necessary. The AOX is nuclear encoded by a small gene family composed of genes in two subfamilies, *AOX1* and *AOX2* [49]. The number of members and the pattern of subfamily ramification are highly dependent on the plant family/species. Plants belonging to the Fabaceae family usually exhibit a single *AOX1* member and at least two *AOX2*-subfamily members (see review in [49] seen in Table 12.1). The first report on *AOX* gene family composition in *P. sativum* is from Sweetman and co-workers [50], showing a single *AOX1*-subfamily member (*PsAOX1*) and two *AOX2*-subfamily members, named *PsAOX2a* and *PsAOX2d*, but no further details are given regarding gene structure and sequence analysis. The analysis performed in the present study confirmed the composition of the *AOX* gene family in *P. sativum* and provided the first report of the structure of each gene. Sequence analysis also revealed a common gene structure in both *PsAOX2*-subfamiy members, with both having a four-exon structure. In contrast, *PsAOX1* has only three exons due to the loss of intron 2. Events of intron loss/gain are responsible for *AOX* gene structure modification and consequent changes in exon sizes (reviewed by [51]). As a consequence of this intron loss, the *PsAOX1* exhibits a 596 bp exon 2 size. Alongside this intron 2 loss, the alignment in Figure 3 reveals the absence of the well-conserved CysI motif located at the N-terminal region of the protein in plants because of an alternative splicing (AS) of intron 1. In plants, the CysI could be replaced by SerI with consequences on enzyme regulation [52]. The absence of CysI could have a strong impact on redox control of enzyme activity [53]. Considering the gene expression pattern observed in cv. ‘Maravilha d’América’ and the difference of transcript accumulation when compared with cv. ‘Torta de Quebrar’, it will be very interesting to further confirm the absence of CysI in both cultivars.

After the identification of the *PsAOX* sequences and structure, the expression was studied in both cultivars during the 16 h imbibition period that activates/initiates germination. Gene expression analysis showed the involvement of the different *PsAOX* gene members at early germination stages in both pea cultivars. Despite exhibiting a similarity in the expression pattern, the influence of the genotype is visible when comparing the level of transcript accumulation. In cv. ‘Maravilha d’América’, in the three *PsAOX* genes, the *PsAOX1* appears as the most responsive gene (peak of up-regulation is seen at 12 hpi in CT with 12 fold-change in comparison with 0 hpi, and at 16 hpi in RT with 17 fold-change). In cv. ‘Torta de Quebrar’, the *PsAOX1* was also the most responsive gene, exhibiting the peak in both CT and RT earlier than in ‘Maravilha d’América’, but with a lower level of transcript accumulated. In CT, the peak was seen at 8 hpi (3 fold-change in comparison with 0 hpi), and in RT, it was seen at 12 hpi (4 fold-change in comparison with 0 hpi). Differential gene expression seen between the two treatments (CT and RT) were confirmed via Western Blotting, which exhibited higher levels of protein in cv. ‘Maravilha d´América’ when compared to cv. ‘Torta de Quebrar’ in both CT- and RT-treated seeds.

A study carried out on the involvement of *AOX* genes in a post-germination phase of *H. perforatum* L. showed that the *HpAOX1* presented an increase in expression throughout the studied period, with the maximum expression level observed 15 days after seed culture, when seedlings were already developed [54]. The differential expression of *AOX1* members in these two species suggests its involvement in different biological processes, seed germination, and seedling development. The involvement of AOX members in developmental processes has been described in several species and is mainly associated with *AOX2*-subfamily members, while AOX1-members have been more associated with plant response to a diversity of stress conditions [25,55]. In the present study, the *PsAOX2a* in both cultivars exhibited a down-regulation pattern independent of the treatment (CT- and RT-treated seeds). To gain insights about AOX involvement in seedling development, further studies need to be carried out during the post-germination phase.

It is interesting that the most responsive genes (*PsAOX1* and *PsAOX2d*) presented a similar pattern, with an increase in transcript level that peaked between 8 and 12 hpi (in *PsAOX1*, only seen under CT) and decreased later in the experiment. The coordinated expression pattern of both genes suggests a co-expression, probably linked to stress response. A study focused on the development of adventitious roots in *Olea europaea* L. (olive) associated the overexpression of *AOX1* subfamily genes (*OeAOX1a* and *OeAOX1d*), seen 8 h after application of the rooting induction stimulus, with the stress caused by preparation of the microcutting, the wooding, and the use of an auxin to induce the morphogenic biological process [56]. In the same way, Campos and colleagues [25] also described a peak of up-regulation in the single-carrot *AOX2*-subfamily (*DcAOX2*) 8 h after callogenesis induction using an in vitro system established with carrot root tissues. The association of increased expression of *PsAOX1* and stress response may be related to the re-establishment of cellular homeostasis in relation to reactive oxygen species (ROS).

Reactive oxygen species are signaling molecules (e.g., superoxide anion, hydrogen peroxide), rapidly released by cells in response to diverse stimuli, which are responsible for long-distance activation of specific metabolic pathways. This phenomenon, called a respiratory burst (reviewed by [57]), must be efficiently controlled in order to avoid cell damage or death. The synthesis of antioxidant molecules (non-enzymatic) and/or the activation of antioxidant enzymes represent the ROS-scavenging mechanisms present in plant cells. The involvement of AOX in restoring a balance of ROS has been demonstrated in different plant species in response to different stress conditions [26,56]. In fact, when the cytochrome pathway is inhibited, ROS accumulation and consequent *AOX1* expression has been observed [58,59]. In the present research, the increase in ROS also seems to be correlated with *PsAOX* expression during pea seed germination. In seeds of cv. ‘Maravilha d´América’, the *PsAOX2d* exhibited a peak of expression at 8 hpi when imbibed with water or RT, coincident with the first peak of ROS accumulation; at 16 hpi, ROS accumulation reached the highest level; that is, the same time point at which *PsAOX1* shows significantly higher values when imbibed in RT in comparison with CT.

The observed relationship between the level of ROS and the expression of *PsAOX* genes appears to be genotype dependent. While cv. ‘Maravilha d’América’ exhibited well-correlated ROS and *PsAOX* expression, cv. ‘Torta de Quebrar’ did not show a clear correlation. Alongside ROS, other molecules could be involved in the regulation of *AOX* genes, which could explain the absence of a link between the level of ROS and *PsAOX* expression. Gray and co-authors [59] demonstrated that TCA cycle intermediates can induce a rapid increase in *AOX1* without a significant increase in ROS levels. Contrastingly, other treatments capable of inducing *AOX1* expression resulted in the generation of significant levels of ROS, which means that two separate pathways for mitochondria-to-nucleus signalling of *AOX1* may exist, one involving ROS and the other organic acids [60]. Furthermore, the enzymatic antioxidant defense mechanism that scavenges and eliminates ROS in plants is composed of various enzymes besides AOX, such as superoxide dismutase (SOD), ascorbate peroxidase (APX), and catalase (CAT) [60].

The cv. ‘Maravilha d´América’ presented higher levels of ROS than cv. ‘Torta de Quebrar’ at all-time points, independently of the treatment, being statistically significant only at the end of germination. The regulation of ROS seems to differ between stress-sensitive and tolerant cultivars [61,62]. Leaves of the tolerant cultivars showed lower ROS production than plants from the sensitive cultivars when submitted to drought and salt stress. The authors attribute this result to the activation of a stronger antioxidant defence in tolerant cultivars [61], which could be similar in the studied pea cultivars.

## 5. Conclusions

In summary, we have reported, for the first time, the involvement of the three *PsAOX* genes in seed germination at both the gene and protein expression levels. Moreover, the germination assay with COX and AOX inhibitors also provided valuable information on the involvement of the alternative pathway in the early stages of pea seed germination. However, monitoring calorespirometric parameters did not provide clear information on the involvement of the AOX pathway but was useful for discriminating among cultivars. It remains to be determined whether monitoring seed germination through calorespirometric measurements at different timepoints would yield different results.

## Figures and Tables

**Table 1:** Percentage of germinated seeds (mean ± standard error) achieved six days after imbibition in three different solutions—SHAM (salicylic hydroxamic acid), Rotenone (RT), and water (control, CT)—for the cvs. ‘Torta de Quebrar’ and ‘Maravilha d’América’. Seeds were incubated under dark conditions, 70% relative humidity, and 25 °C temperature for six days. Different letters indicate significant differences between inhibitor treatments and cultivars according to Dunn test. Statistical significance was considered for *p* ≤ 0.05.

Cultivar	Treatment		
	Water	Rotenone	SHAM
Torta de Quebrar	73 ± 3.2 d	46 ± 5.6 c	5 ± 2.7 a
Maravilha d’América	56 ± 3.1 cd	29 ± 3.7 bc	14 ± 3.9 ab

**Table 2:** *p*-values of the two-way ANOVA for the effects of the cultivar and inhibitor treatment, as well as their interaction in respiratory heat rate (Rq), CO_2_ production rate (R_CO_2__), structural biomass growth rate (Rstruct_bio), and substrate carbon conversion efficiency (ε). Statistical significance was considered for *p* ≤ 0.05.

Effect	*p*-Values			
	R_q_	R_CO_2__	R_struct_bio_	ε
Cultivar	<0.001	<0.001	<0.001	0.020
Treatment	0.920	0.762	0.931	0.894
Cultivar × Treatment	0.384	0.523	0.683	0.515

**Table 3:** *p*-values of the three-way ANOVA for the effects of the cultivar and treatment, as well as their interaction in *PsAOX* gene expression. Statistical significance was considered for *p* ≤ 0.05.

Effect	*p*-Values		
	*PsAOX1*	*PsAOX2a*	*PsAOX2d*
Cultivar	<0.001	<0.001	0.001
Treatment	0.091	0.052	0.003
Imbibition period (Ip)	<0.001	<0.001	<0.001
Cultivar × Treatment	0.306	0.898	0.948
Cultivar × Ip	<0.001	0.003	<0.001
Treatment × Ip	<0.001	0.007	0.368
Cultivar × treatment × Ip	<0.001	0.125	0.023

**Table 4:** *p*-values of the two-way ANOVA for the effects of the inhibitor treatment and imbibition period, as well as their interaction in PsAOX expression. Statistical significance was considered for *p* ≤ 0.05.

Effect	*p*-Values
	Total Protein
Cultivar	0.003
Treatment	0.502
Imbibition period (Ip)	0.343
Cultivar × Treatment	0.058
Cultivar × Ip	<0.001
Treatment × Ip	0.080
Cultivar × Treatment × Ip	0.014

**Table 5:** (**a**) *p*-values of the three-way ANOVA to study the effects of the cultivar, inhibitor treatment, imbibition period, as well as their interaction in total ROS levels. Statistical significance was considered for *p* ≤ 0.05. (**b**) Mean values of four replicates ± standard error for the different groups of each factor (cultivar, treatment, and imbibition period). Different letters next to each value indicate significant differences according to Dunn test.

(a) Effects (ANOVA)	*p*-Values
Cultivar	<0.001
Treatment	0.259
Imbibition period (Ip)	<0.001
Cultivar × Treatment	0.135
Cultivar × Ip	0.065
Treatment × Ip	0.089
Cultivar × Treatment × Ip	0.468
**(b) Factor**	**RFU**
**Cultivar**	
Maravilha d´América	496.22 ± 71.336 a
Torta de Quebrar	828.52 ± 108.3 b
**Treatment**	
Non-treated (CT)	625.28 ± 104.050 a
Rotenone-treated	664.69 ± 106.005 a
**Imbition period**	
0 h	331.6 ± 30.518 a
4 h	457.58 ± 58.683 ab
8 h	855.21 ± 107.51 cd
12 h	677.72 ± 102.414 bc
16 h	989.74 ± 198.017 d

## Data Availability

Not applicable.

## Supplementary Materials

The following supporting information can be downloaded at https://www.mdpi.com/article/10.3390/biology12101318/s1: Figure S1: Experimental design; Figure S2: Gene structure of *AOX* genes from *Pisum sativum* (Psat2g sequences) in comparison with *AOX* genes from plant species belonging to Fabaceae family (Gm—*Glycine max*, Mt—*Medicago trucatula*, Phv—*Phaseolus vulgaris*, Tp—*Trifolium pratense*, and Vigun—*Vigna unguiculata)*. Genomic and transcript sequences were retrieved from Ensembl Plants database (https://plants.ensembl.org/index.html). Schematic was made by using GSDS 2.0 software (available at http://gsds.gao-lab.org/). Exons are depicted in blue, and introns are represented by black lines; Figure S3: Representative Western blot membrane of AOX and Actin in seeds collected at 4 h post imbibition in water (Control—C) in both cultivars, the cv. ‘Maravilha d’América’ (MA) and cv. ‘Torta de Quebrar’ (TQ). For each cultivar, condition, and time point, there were four biological replicates (C1, C2, C3, and C4); Table S1: Eudicot plant species used in the NJ analysis; Table S2: Monocot plant species used in the NJ analysis. In grey the AOX2 sequence; Table S3: Primers used in gene expression analysis (reference genes: PsPOB and PsSAR1B, and target genes: PsAOX1, PsAOX2a and PsAOX2b) and some parameters related with amplification. Primers were designed using the software Primer3Plus, freely available at link https://primer3plus.com/cgi-bin/dev/primer3plus.cgi, accessed 17 April 2021; Statistical significance was considered for *p* ≤ 0.05.
