# Supplementary material for: Germination of Pisum sativum L. Seeds Is Associated with the Alternative Respiratory Pathway
Source: Biology (Basel). 2023 Oct 9;12(10):1318. doi: 10.3390/biology12101318 (PMC10604721; doi:10.3390/biology12101318)
Supplement: Supplementary file 1 [file biology-12-01318-s001.zip › Table S1.pdf]

Table S1. Eudicot plant species used in the NJ analysis.

O	F	Species	Accession number
Brassicales	*Brassicaceae*	*Arabidopsis lyrata*	AL1G33660<br>AL3G24680<br>AL3G24690<br>AL5G06730<br>AL0G08000<br>AL8G30860
		Arabidopsis thaliana	AT1G32350<br>AT3G22360<br>AT3G22370<br>AT3G27620<br>AT5G64210
		Boechera stricta	Bostr.19424s0421.1<br>Bostr.19424s0422.1<br>Bostr.3359s0124.1<br>Bostr.0568s0383.1
		Brassica rapa	Bra010153<br>Bra001865<br>Bra031351<br>Bra023835<br>Bra037768
		Capsella grandiflora	Cagra.1189s0011.1<br>Cagra.1189s0012.1<br>Cagra.5575s0007.1<br>Cagra.3957s0019.1<br>Cagra.0248s0102.1
		Capsella rubella	Carubv10014065m<br>Carubv10019557m<br>Carubv10009739m<br>Carubv10026681m
		Eutrema salsugineum	Thhalv10021013m<br>Thhalv10009342m<br>Thhalv10005697m
		<u>*Thellungiella parvula*</u>	TP3G20130<br>TP3G20140<br>TP2G27270
	Ca ric	*Carica papaya*	evm.model.supercontig_8.29<br>evm.model.supercontig_42.47
Curcubi	*Curc*	<u>*Citrullus lanatus*</u>	CL10G07420
		<u>*Cucumis melo*</u>	CM00136G00010
		Cucumis sativus	Cucsa.398150.1
Fabales	*Fabaceae*	*Glycine max*	GM04G14800<br>GM08G07690<br>GM08G07700<br>GM05G24455
		Lotus japonicus	LJ2G020780<br>LJ4G005280<br>LJ4G005290
		Medicago truncatula	MT5G026620<br>MT5G070680

O	F	Species	Accession number
			MT5G070870<br>MT5G070880
		Phaseolus vulgaris	Phvul.002G127100.1<br>Phvul.002G209100.1<br>Phvul.002G209200.1
Malvales	*Malvaceae*	*Gossypium raimondii*	Gorai.008G296600.1<br>Gorai.012G142200.1<br>Gorai.005G220400.1<br>Gorai.005G220500.1
		Theobroma cacao	TC03G031300<br>TC02G011670
Malpighiales	*Linace ae*	*Linum usitatissimum*	Lus10035670<br>Lus10005372<br>Lus10020523
	Salicaceae	*Populus trichocarpa*	PT03G09340<br>PT12G01430<br>PT12G01440<br>PT15G01960
		Salix purpurea	SapurV1A.1470s0080.1<br>SapurV1A.0346s0170.1<br>SapurV1A.0377s0140.1<br>SapurV1A.0377s0150.1<br>SapurV1A.3352s0030.1<br>SapurV1A.0894s0160.1
	Eu pho	*Manihot esculenta*	ME10292G00060
		Ricinus communis	RC30063G00030
	H	*Hypericum perforatum*	EU330415.1<br>EU330413.1
Ranuncul	*R*	*Aquilegia coerulea Goldsmith*	Aquca_105_00003.1<br>Aquca_043_00024.1<br>Aquca_008_00134.1<br>Aquca_033_00110.1
Rosales	*Rosaceae*	*Fragaria vesca*	FV5G29310<br>FV5G21950
		Malus domestica	MD00G028680<br>MD00G081720<br>MD13G026910<br>MD16G016620
		Prunus persica	Prupe.5G018700.1<br>Prupe.1G061800.1<br>Prupe.1G061900.1<br>Prupe.1G061400.1
Sapindales	*Rutaceae*	*Citrus clementina*	Ciclev10001766m<br>Ciclev10003687m<br>Ciclev10028835m
		Citrus sinensis	orange1.1g037339m<br>orange1.1g019765m<br>orange1.1g020532m
Solanales	*Solanaceae*	*Solanum lycopersicum*	Solyc08g005550<br>Solyc08g075540<br>Solyc08g075550<br>Solyc01g105220
		Solanum tuberosum	PGSC0003DMT400019708<br>PGSC0003DMT400019707<br>PGSC0003DMT400047562

O	F	Species	Accession number
			PGSC0003DMG400012558
A	*a*	*Amborella trichopoda*	ATR_00038G01180<br>ATR_00048G01570
B	*b*	*Beta vulgaris*	BV5G19180<br>BV9G03180
C	*c*	*Kalanchoe laxiflora*	Kalax.0453s0008.1<br>Kalax.1476s0004.1<br>Kalax.0496s0017.1<br>Kalax.0907s0011.1<br>Kalax.0414s0014.1
D	*d*	*Mimulus guttatus*	Migut.J01127.1<br>Migut.E01358.1<br>Migut.N01067.1
E	*e*	*Eucalyptus grandis*	Eucgr.E01214.1<br>Eucgr.E01213.1<br>Eucgr.I02663.1
F	*f*	*Vitis vinifera*	VV02G09030<br>VV02G09050<br>VV00G00110

O: order, F: family; C: *Caricaceae*, Curcubi: *Curcubitales*, Curc: *Curcubitaceae*, Lina: *Linaceae*, E: *Euphorbiaceae*, Ranun: *Ranunculales*, R: *Ranunculaceae*, A: ***Amborellales,*** B: *Caryophyllale*, C: *Saxifragales*, D: Lamiales, E: Myrtales, F: *Vitales*, a: ***Amborellaceae***, b: *Amarantaceae*, c: *Crassulaceae*, d: *Phrymaceae*, e: *Myrtaceae*, f: *Vitaceae*. In grey the AOX2 sequences.
