# Supplementary material for: Germination of Pisum sativum L. Seeds Is Associated with the Alternative Respiratory Pathway
Source: Biology (Basel). 2023 Oct 9;12(10):1318. doi: 10.3390/biology12101318 (PMC10604721; doi:10.3390/biology12101318)
Supplement: Supplementary file 1 [file biology-12-01318-s001.zip › Table S2.pdf]

Table S2. Monocot plant species used in the NJ analysis. The AOX2 sequence is in grey.

Species	Accession number
Brachypodium distachyon	BD3G52505<br>BD5G20540<br>BD5G20547<br>BD5G20557
Brachypodium stacei	Brast04G094100.1<br>Brast09G194700.1<br>Brast09G194600.1<br>Brast09G194800.1
Hordeum vulgare	CAJW010038523<br>CAJW011587016<br>CAJW010099492
Musa acuminata	GSMUA_Achr5G03810_001<br>GSMUA_Achr6G01170_001<br>GSMUA_Achr6G01300_001<br>GSMUA_Achr1G27800_001
Oryza brachyantha	OB02G22630<br>OB02G36280<br>OB04G30980<br>OB04G30990
Oryza glaberrima	ORGLA02G0249500<br>ORGLA04G0206000<br>ORGLA04G0206100
Oryza sativa	BGIOSGA008063<br>BGIOSGA005788<br>BGIOSGA014421<br>BGIOSGA014422
Panicum hallii	Pahal.G02176.1<br>Pahal.G02177.1<br>Pahal.G02175.1<br>Pahal.A03053.1
Panicum virgatum	Pavir.Ab01160.1<br>Pavir.Aa00784.1<br>Pavir.Ga00730.1<br>Pavir.Gb00786.1<br>Pavir.Ga00729.1<br>Pavir.Ga00625.1<br>Pavir.Ab02811.1<br>Pavir.Gb00785.1<br>Pavir.Gb00789.1
Setaria italica	Seita.7G223800.1<br>Seita.7G223900.1<br>Seita.7G223700.1<br>Seita.1G286500.1
Setaria viridis	Sevir.7G235500.1<br>Sevir.7G235600.1<br>Sevir.7G235400.1<br>Sevir.1G291700.1
Spirodela polyrhiza	Spipo20G0025000<br>Spipo5G0073900<br>Spipo11G0008300
Sorghum bicolor	SB04G030820<br>SB06G027410<br>SB06G027420<br>SB06G027430
Zea mays	ZM02G05480<br>ZM02G05490<br>ZM02G05500<br>ZM05G37570
