# Supplementary material for: Germination of Pisum sativum L. Seeds Is Associated with the Alternative Respiratory Pathway
Source: Biology (Basel). 2023 Oct 9;12(10):1318. doi: 10.3390/biology12101318 (PMC10604721; doi:10.3390/biology12101318)
Supplement: Supplementary file 1 [file biology-12-01318-s001.zip › Table S3.pdf]

Table S3. *Primers* used in gene expression analysis (reference genes: *PsPOB* and *PsSAR1B*, and target genes: *PsAOX1*, PsAOX2a and *PsAOX2b*) and some parameters related with amplification. Primers were designed using the software Primer3Plus, freely available at link https://primer3plus.com/cgi-bin/dev/primer3plus.cgi [71].

Gene	Accession	Primers 5′-3′	[primer] (nM)	T_a (ºC)	AL (bp)	E(%)	R^2
PsPOB	Psat6g143120.1	Fw: TGGGTGGTGGATTCGGATTT<br>Rv: TGCTCGAAATTTGGCAAGGC	400	62	100	103.4	0.999
PsSAR1B	Psat3g012960.1	Fw: TTTGGGCCTGAGCAACTTCA<br>Rv: TACCTCCATGGGACGGACAT	300	60	95	104	0.988
PsAOX1	Psat2g142680.1	Fw: GCACTTTTGCGGCCAATTTG<br>Rv: GCACCAGCACTACCTTGAGA	400	60	67	99.5	0.994
PsAOX2d	Psat2g058520.1	Fw: AACAGCAAAGCCGTCGTTTC<br>Rv: CCATGGCCACTCAGTTCCAT	300	60	56	100.3	0.999
PsAOX2a	Psat7g098200.1	Fw: TACGAGAGAGGATGGGACGG<br>Rv: AAGTCTCCCACGGCATGAAG	300	60	81	93	0.999

T_a: *annealing* temperature, AL: amplicon length, E: efficiency, R^2: coefficient of linear regression.
