# Supplementary figures and images for: Germination of Pisum sativum L. Seeds Is Associated with the Alternative Respiratory Pathway
Source: Biology (Basel). 2023 Oct 9;12(10):1318. doi: 10.3390/biology12101318 (PMC10604721; doi:10.3390/biology12101318)

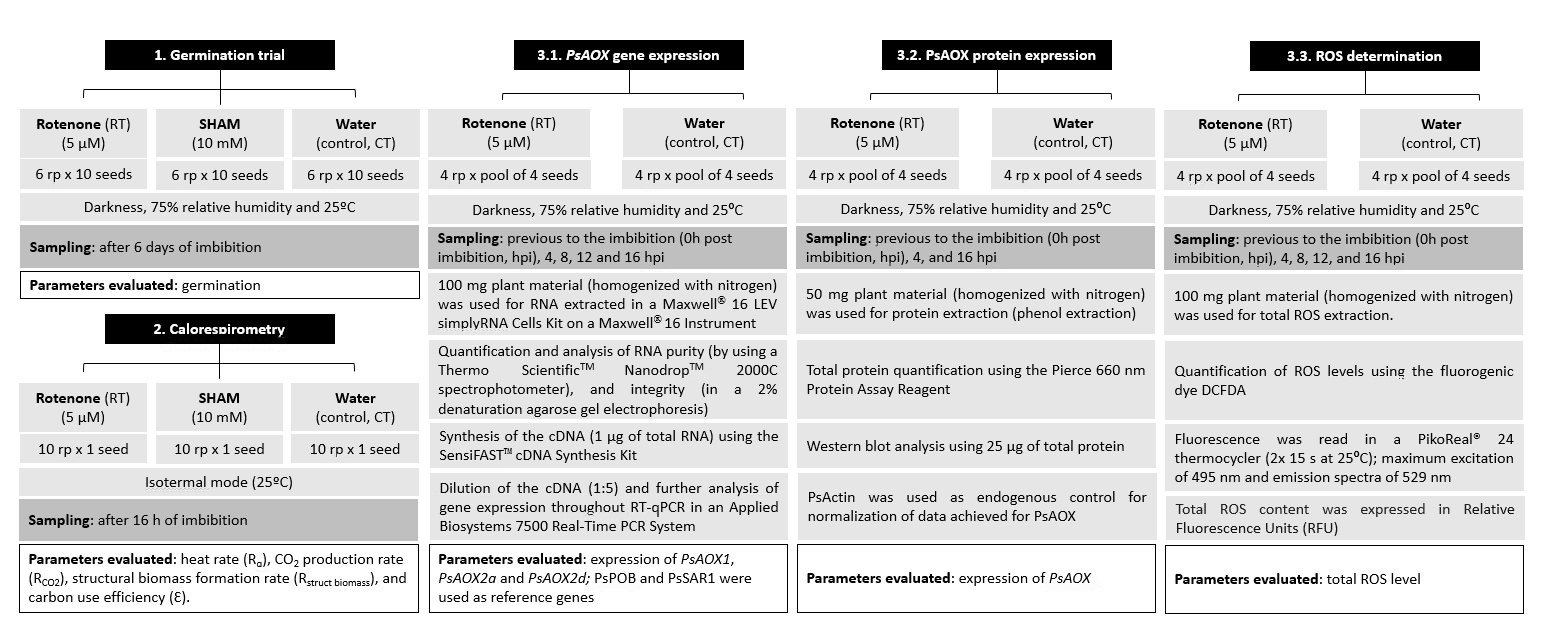

Supplement: Supplementary file 1 [file biology-12-01318-s001.zip › FigureS1.jpg]

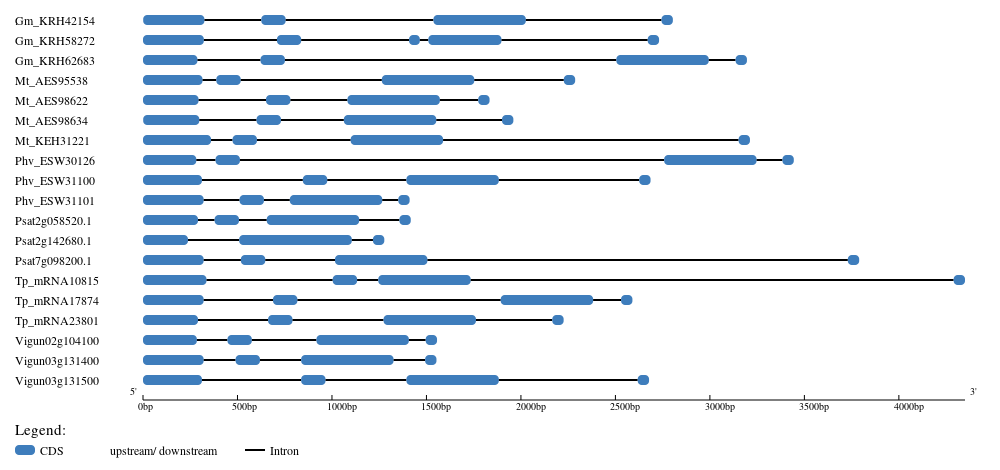

Supplement: Supplementary file 1 [file biology-12-01318-s001.zip › FigureS2.jpg]

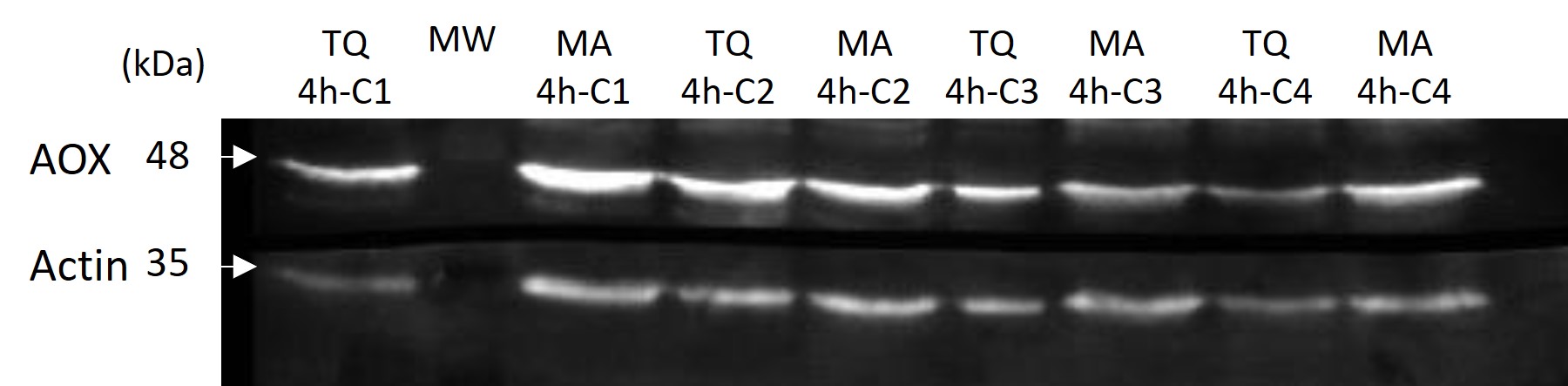

Supplement: Supplementary file 1 [file biology-12-01318-s001.zip › FigureS3.jpg]
